# Clinical utility of ultra-widefield fluorescein angiography and optical coherence tomography angiography for retinal vein occlusions

**DOI:** 10.3389/fmed.2023.1110166

**Published:** 2023-06-08

**Authors:** Tien-En Tan, Farah Ibrahim, Priya R. Chandrasekaran, Kelvin Yi Chong Teo

**Affiliations:** ^1^Singapore Eye Research Institute, Singapore National Eye Centre, Singapore, Singapore; ^2^Duke-NUS Medical School, Singapore, Singapore

**Keywords:** branch retinal vein occlusion (BRVO), central retinal vein occlusion (CRVO), ultra-widefield (UWF), optical coherence tomography angiography (OCTA), fluorescein angiography (FA), retinal imaging, imaging modality

## Abstract

Retinal vein occlusions (RVOs) are the second most common retinal vascular disease after diabetic retinopathy, and are a significant cause of visual impairment, especially in the elderly population. RVOs result in visual loss due to macular ischemia, cystoid macular edema (CME), and complications related to neovascularization. Vascular assessment in RVOs traditionally relies on standard fluorescein angiography (FA) for assessment of macular and retinal ischemia, which aids in prognostication and guides intervention. Standard FA has significant limitations—it is time-consuming, requires invasive dye administration, allows for limited assessment of the peripheral retina, and is usually evaluated semi-qualitatively, by ophthalmologists with tertiary expertise. More recently, the introduction of ultra-widefield FA (UWF FA) and optical coherence tomography angiography (OCTA) into clinical practice has changed the tools available for vascular evaluation in RVOs. UWF FA allows for evaluation of peripheral retinal perfusion, and OCTA is non-invasive, rapidly-acquired, and provides more information on capillary perfusion. Both modalities can be used to provide more quantitative parameters related to retinal perfusion. In this article, we review the clinical utility and impact of UWF FA and OCTA in the evaluation and management of patients with RVOs.

## 1. Introduction

Retinal vein occlusions (RVOs) are the second most common retinal vascular disease after diabetic retinopathy (DR), and are a significant cause of visual impairment, especially in the elderly population. Globally, it was estimated in 2015 that RVO affects 28 million individuals, with 0.77% prevalence among individuals aged 30–89 years (1). Broadly, there are two main types of RVOs: central retinal vein occlusion (CRVO) occurs due to an occlusion (usually thrombotic) of the central retinal vein at or posterior to the lamina cribrosa, while a branch retinal vein occlusion (BRVO) occurs at the level of a retinal venule, usually at the site of an arteriovenous crossing (2). BRVOs are about 5 times more common than CRVOs, but both of these subtypes share common major risk factors (1–3). The factors leading to thrombosis and RVO follow that of Virchow’s triad, namely vascular wall or endothelial damage, venous stasis, and hypercoagulability. Accordingly, the major risk factors for most RVOs, which are older age, hypertension, hyperlipidemia, diabetes mellitus and cigarette smoking, are all related to vascular endothelial damage, and therefore intricately linked to systemic vascular disease as well (2). Consequently, upon diagnosis of an RVO, systemic screening and management of these underlying systemic vascular diseases is crucial to prevent an RVO recurrence, as well as other related atherosclerotic diseases such as coronary artery disease or cerebrovascular accidents.

From an ocular perspective, visual loss in RVOs usually occurs due to macular ischemia, cystoid macular edema (CME), or complications of neovascularization such as vitreous hemorrhage, tractional retinal detachment and neovascular glaucoma (NVG). Various forms of retinal imaging play a crucial role in detecting and guiding management of these ocular complications. Optical coherence tomography (OCT) is the modality of choice for diagnosis of CME, and CME can be treated with intravitreal anti-vascular endothelial growth factor (anti-VEGF) injections, intravitreal corticosteroid injections, or macular grid laser photocoagulation in select cases (4–7). Fluorescein angiography (FA) is the traditional standard of care imaging modality for determining risk of neovascularization after RVOs, and areas of retinal non-perfusion quantified by standard FA are the basis for classification of RVOs as “ischemic” or “non-ischemic.” Specifically, the landmark Branch Vein Occlusion Study (BVOS) defined “ischemic” BRVOs as those with at least five disc areas of retinal non-perfusion, and demonstrated that such eyes had a 40% risk of developing retinal neovascularization at 3 years (8). Similarly, the Central Vein Occlusion Study (CVOS) defined “ischemic” CRVOs as those with at least 10 disc areas of retinal non-perfusion. “Ischemic” or indeterminate CRVOs had a 35% risk of developing iris neovascularization at 3 years, compared to “non-ischemic” eyes that had only a 10% risk (9). Scatter laser photocoagulation is effective in preventing neovascularization and vitreous hemorrhage in BRVOs, and in inducing regression of iris neovascularization in CRVOs, and therefore, FA is crucial in clinical practice for prognostication of RVOs, and for guiding clinical management (8, 10). However, FA has some important limitations. First, FA requires the administration of fluorescein dye, which is invasive, time-consuming, and carries some systemic risk, including allergy, anaphylaxis, and cardiac events (11). Second, “standard” FA is typically performed with fundus cameras capable of 30°–55° fields of view. Even with montage of steered images, this provides assessment of the posterior pole and up to the mid-periphery only, with limited assessment of the retinal far periphery (12). Third, evaluation and assessment of standard FA is typically performed qualitatively or semi-quantitatively, and requires tertiary retinal specialist expertise.

New imaging technologies have been introduced recently, that significantly improve our ability to evaluate the retinal vasculature, and can overcome many of the challenges associated with standard FA imaging. Ultra-widefield (UWF) imaging technology allows for a much wider field of view and reproducible, objective assessment of the retinal far periphery (12). Optical coherence tomography angiography (OCTA) now allows for non-invasive evaluation of the retinal vasculature, including evaluation of the retinal capillary microvasculature, without the need for invasive dye (13). These imaging technologies are commercially available and increasingly accessible, and they have transformed how we clinically assess the retinal vasculature in RVOs, as well as other retinal vascular diseases. In this paper, we review the utility and impact of UWF FA and OCTA in the clinical assessment, evaluation, and management of RVOs.

## 2. Ultra-widefield fluorescein angiography

### 2.1. Ultra-widefield imaging

**Figure 1:**
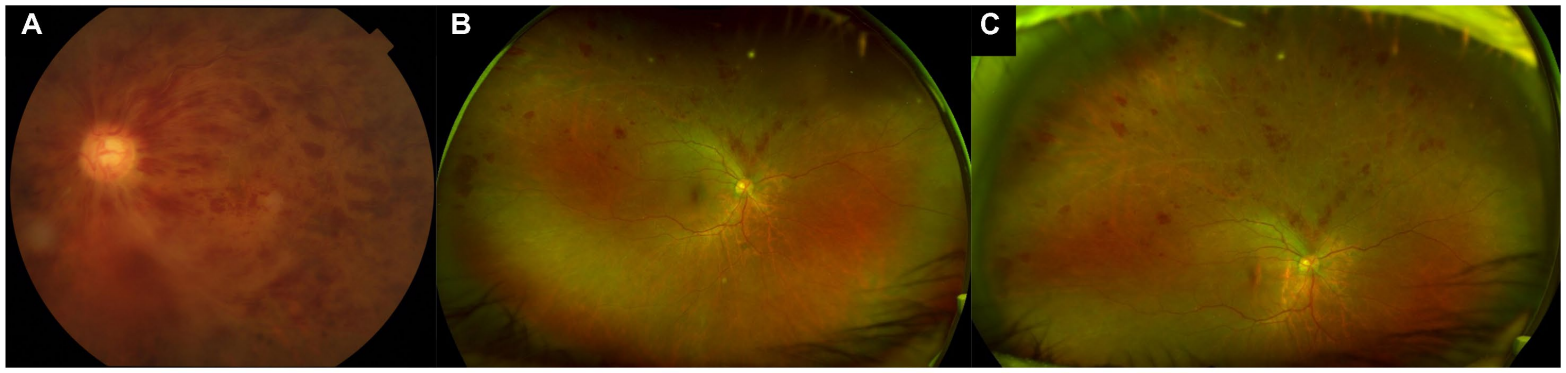
Standard color and ultra-widefield (UWF) photographs of retinal vein occlusions. **(A)** Standard 50° color photograph of a central retinal vein occlusion, allowing for visualization just beyond the posterior pole. **(B)** Ultra-widefield (UWF) pseudocolor photograph of a superior branch retinal vein occlusion, showing a much wider field of view, including much of the retinal periphery. **(C)** Steered UWF pseudocolor photograph of the same eye as in **(B)**, but taken in upgaze, to provide a better view of pathology in the superior far periphery.

Imaging techniques for assessment of retinal vascular disease have evolved significantly over the years. For example, in DR severity grading and assessment, the established gold standard for the past three decades has been the Early Treatment of Diabetic Retinopathy Study (ETDRS) fundus photography protocol, which originally captured seven standardized 30° fields of the posterior retina with film-based cameras (14). These standard photographic fields represented the area of the retina that could be reliably and reproducibly imaged at the time. However, in total, these seven fields only cover about 30% of the retinal surface area (15, 16). We have since transitioned to digital photographs and larger fields of view, but standard fundus cameras still generally have fields of view between 45° and 55°, which limits assessment of the retinal far periphery. More recent advances in UWF fundus imaging technology have enabled reliable imaging of the retinal far periphery, with commercial UWF imaging platforms such as the Optos (Optos PLC, Dunfermline, United Kingdom) able to capture up to 200° in a single image, which corresponds to about 80% of the retina (Figure 1) (15). Montage of steered images increases the field of view to 220°, covering more than 95% of the retina. Terminology for such imaging capability has been variable, but a recent consensus statement amongst retinal imaging experts has defined UWF imaging as having a field of view of 110°–220°, and providing visualization of at least the anterior edge of the vortex vein ampullae and beyond (12).

The more extensive view of the peripheral retina that is provided by UWF imaging allows for detection of additional peripheral lesions, which have important implications for diagnosis, prognostication, and management of various retinal vascular diseases (Figure 1). For example, in DR, the use of UWF imaging detects a more severe level of DR compared to standard ETDRS 7-field photographs in 10%–19% of eyes (17–20). Furthermore, the presence of predominantly peripheral lesions (PPLs) and areas of non-perfusion on UWF FA in DR have been identified as significant independent risk factors for DR progression, above and beyond the risk stratification provided by the ETDRS severity scale grading (21, 22). Clearly, there is valuable information in the retinal periphery that can help to inform our clinical management in patients with retinal vascular disease.

### 2.2. UWF FA and risk of neovascularization

**Figure 2:**
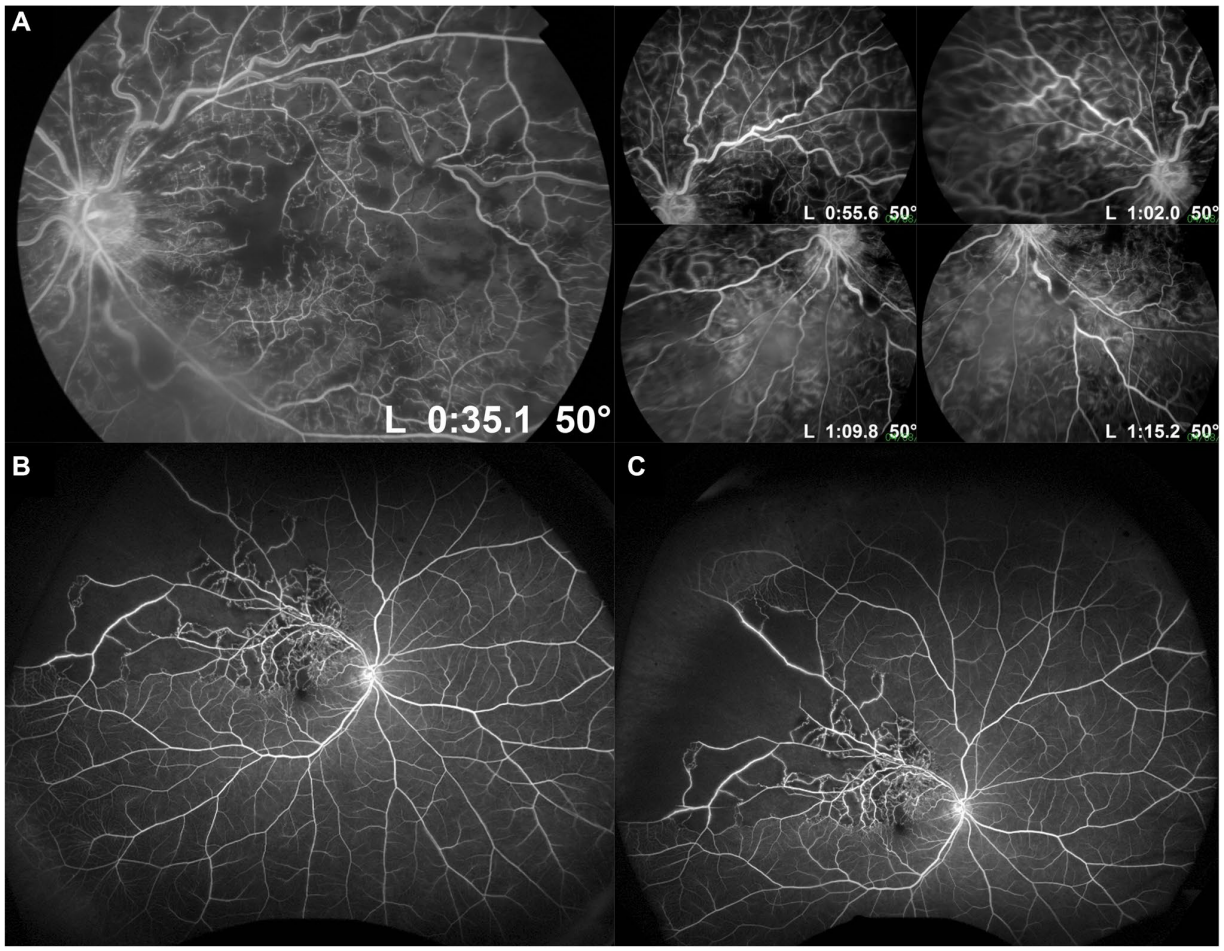
Standard and ultra-widefield (UWF) fluorescein angiography (FA) of retinal vein occlusions. **(A)** Standard 50° FA images of a central retinal vein occlusion in primary gaze showing the posterior pole, and steered in different directions of gaze to increase the field of view up to the mid-periphery. **(B)** UWF FA image of a superotemporal branch retinal vein occlusion, showing a much wider field of view, including much of the retinal periphery. **(C)** Steered UWF FA image of the same eye as in **(B)**, but taken in upgaze to provide a better view of pathology in the superior far periphery. This image shows extensive peripheral retinal non-perfusion out to the superotemporal far periphery, which would not have been appreciated on standard FA.

Similarly, UWF FA imaging can provide important information from the retinal periphery in the clinical assessment of eyes with RVO. The disease process in RVOs affects the peripheral retina as well as the posterior retina. Retinal non-perfusion and ischemia in RVOs is not confined to the posterior pole, and frequently extends out to the far periphery, based on the area of retina drained by the occluded venule (Figure 2). Turczyńska et al. demonstrated in 102 eyes with RVO that 59.8% had zones of peripheral ischemia (23). Such areas of retinal non-perfusion are a significant risk factor for iris and retinal neovascularization, which can lead to visual loss (8, 9, 24). Standard FA frequently underestimates the amount of retinal non-perfusion that is present, and in some cases can completely miss areas of peripheral non-perfusion. For example, in a retrospective series of 42 CRVO cases imaged with UWF FA, Nicholson et al. found that 31.0% of eyes had significant peripheral non-perfusion, despite a completely perfused posterior pole (25). Similarly, in the series by Turczyńska et al., 20.6% of eyes showed only peripheral ischemia, with no detectable ischemia in the posterior pole. Such peripheral ischemia was only visualized on UWF FA, and this was significant as it required a change in treatment decision with scatter laser photocoagulation in their series (23). In clinical practice, with only standard FA, such eyes may be mis-labelled as “non-ischemic,” and may return with visual loss from neovascular complications that could otherwise have been prevented.

By imaging a much larger area of the retina, UWF FA reveals larger absolute areas of non-perfusion and ischemia in RVOs than standard FA (Figure 2). Therefore, our traditional cut-off values of 5 and 10 disc areas for BRVOs and CRVOs, respectively, to be considered “ischemic” need to be re-evaluated (8, 9). Tsui et al. attempted to develop a cut-off value based on an ischemic index (ISI) on UWF FA images (26). ISI was measured by delineating the area of retinal non-perfusion, and dividing it by the total retinal area in pixels, as seen in the arteriovenous phase of a UWF FA image. They suggested an ISI of more than 45% was associated with greater risk of neovascularization. Thomas et al. similarly measured ISI on UWF FA images in a retrospective cohort of 60 eyes, and suggested that an ISI of 35% or more was highly sensitive and specific for ischemic CRVOs (27). However, both these studies did not account for peripheral image magnification on UWF images and distortion correction. Nicholson et al. subsequently addressed this issue of image distortion on UWF FA in their series of 42 eyes with CRVO, and also attempted to develop a clinically-useful cut-off value (25). They found that when considering total retinal non-perfusion on UWF FA, 30 disc areas was probably a more useful cut-off than 10 disc areas: risk of neovascularization was only 5.3% in eyes with less than 30 disc areas of non-perfusion, but increased to 52.2% when this threshold was exceeded. However, their findings also suggested that the distribution of non-perfusion made a difference as well. More than 10 disc areas of non-perfusion in the posterior pole conferred a significantly higher risk of neovascularization than 10 disc areas of non-perfusion in the periphery, if the posterior pole remained perfused. More studies, with longitudinal design, are required to address this question, and to validate clinically-relevant thresholds of non-perfusion on UWF FA for intervention in both CRVOs and BRVOs. Table 1 summarizes the key advantages and disadvantages of UWF FA for detection of retinal non-perfusion and neovascularization in RVOs.

**Table 1:** Advantages and disadvantages of ultra-widefield fluorescein angiography and optical coherence tomography angiography for detection of retinal non-perfusion and neovascularization in retinal vein occlusions.

		UWF FA	OCTA
Detection of retinal non-perfusion	Advantages	Greater field of view, ability to detect much larger areas of non-perfusion, including up to the far periphery, which would be missed on standard FA or OCTA	Non-perfusion areas correlate/agree well with FA, and may be more accurate (no issues with choroidal background fluorescence)
		Peripheral non-perfusion areas linked to risk of neovascularization (but needs further validation)	Non-perfusion areas linked to risk of neovascularization (but needs further validation)
		Greater focus on objective, quantitative parameters (compared to standard FA), such as ISI	Provides automated, objective, quantitative parameters and metrics in relation to perfusion
			No dye leakage to interfere with measurement/quantification of non-perfusion
			Non-invasive, with no systemic risk, and faster, more convenient acquisition of images
	Disadvantages	Clinically-useful cut-offs for non-perfusion have yet to be determined and validated—current evidence mostly cross-sectional, and need for more longitudinal studies	Clinically-useful cut-offs for non-perfusion have yet to be determined and validated—current evidence mostly cross-sectional, and need for more longitudinal studies
		Variability of total area of imaged/gradable peripheral retina can result in changes to ISI and other quantitative metrics	Limited field of view with current technology compared to UWF FA, even with montage of multiple steered images
		Potential inaccuracies related to peripheral image distortion and warp	Heterogeneity and lack of standardization in commercial devices and metrics
		Potential inaccuracies related to changes in choroidal background fluorescence	Image artefacts, quality and gradability
		Potential inaccuracies related to dye leakage, which can obscure areas of non-perfusion	
		Quantitative metrics currently derived manually, which is time-consuming and ill-suited to clinical application	
		Fundamentally similar to standard FA—still requires invasive dye administration, with associated systemic risk, and takes time to acquire	
Detection of neovascularization	Advantages	Greater field of view, allows for detection of neovascularization in retinal periphery, which would be missed on standard FA or OCTA	Better differentiation of collaterals from neovascularization—cross-sectional scans can detect flow/vascular structures anterior to ILM
		Image interpretation potentially easier—no need for segmentation, and greater familiarity for ophthalmologists and retinal specialists	Potentially more sensitive for detection of neovascularization within the same area as FA
			Better structural characterization of neovascular lesions
			Non-invasive, with no systemic risk, and faster, more convenient acquisition of images
	Disadvantages	Detection of neovascularization dependent on leakage, which can be variable or minimal in some cases—can lead to neovascularization being missed	Limited field of view with current technology compared to UWF FA, even with montage of multiple steered images
		Fundamentally similar to standard FA—still requires invasive dye administration, with associated systemic risk, and takes time to acquire	Technically more difficult to interpret, with need for segmentation and scrolling through cross-sectional scans

UWF, ultra-widefield; FA, fluorescein angiography; OCTA, optical coherence tomography angiography; ISI, ischemic index; ILM, internal limiting membrane.

### 2.3. UWF FA and cystoid macular edema

Besides neovascularization, UWF FA abnormalities are also associated with other important complications of RVOs, such as CME. CME is a common complication, and a major cause of visual impairment in RVOs. It has long been postulated that the extent and location of retinal ischemia may be causally related to the development and persistence of CME, due to the upregulation of VEGF and other vasogenic and inflammatory cytokines from areas of non-perfusion (28, 29). These cytokines and mediators result in breakdown of the inner blood-retinal barrier and increased vascular permeability, which lead to the development of CME. Several cross-sectional studies have linked areas of retinal non-perfusion on UWF FA to CME. In a few studies, greater areas of ischemia (quantified by ISI) have been correlated with increasing severity of CME and central macular thickness (30, 31). Prasad et al. examined UWF FA images in 76 eyes with BRVO, and found that areas of untreated non-perfusion were associated with CME. Interestingly, in this study, it was particularly areas of peripheral non-perfusion (anterior to the equator) that were strongly associated with CME, whereas the same association was not evident with posterior non-perfusion (29). It has also been suggested that areas of *partial* retinal ischemia are more likely to be associated with CME than areas of *complete* ischemia (28, 32). It is postulated that in partial ischemia, viable cells are still capable of producing cytokines in response to ischemia, whereas in complete ischemia there is cellular loss, reduced oxygen demand, and hence less cytokine-driven CME. Besides areas of non-perfusion, leakage evident on UWF FA has also been linked to CME. Wang et al. quantified leakage in various retinal zones on UWF FA in eyes with CRVO, and showed that greater leakage indices were significantly associated with the presence of CME. Furthermore, increased central macular thickness was correlated with pan-retinal leakage, peri-macular leakage, near-peripheral leakage, and mid-peripheral leakage, with the strongest correlation for peri-macular leakage (33).

Greater areas of retinal non-perfusion have been linked to RVOs with recalcitrant CME (31). Accordingly, greater areas of retinal non-perfusion on UWF FA are associated with higher anti-VEGF treatment burden for CME (34). Abri Aghdam et al. quantified non-perfusion on UWF FA in treatment-naïve CRVOs with CME, and found that cases with greater non-perfusion at baseline subsequently required significantly more ranibizumab injections (34). Following the hypothesis that recalcitrant CME is driven by persistent areas of untreated retinal non-perfusion, various groups have attempted to use UWF FA to guide targeted scatter laser photocoagulation as an adjunct treatment for CME, with conflicting results. Tomomatsu et al. reported results from a randomized clinical trial comparing intravitreal bevacizumab and targeted laser photocoagulation versus bevacizumab alone for CME in patients with ischemic BRVOs, showing that the addition of laser photocoagulation reduced the burden of bevacizumab retreatments (35). Similarly, Goel et al. showed in another randomized clinical trial for BRVOs with CME that the addition of UWF FA-guided targeted laser photocoagulation reduced the number of intravitreal ranibizumab treatments required (36).

In contrast, the WAVE trial did not demonstrate a significant reduction in ranibizumab injections with targeted laser photocoagulation in RVO patients with CME. This may have been due to the relatively modest sample size in the study (*n* = 30), but they also postulated that the lack of treatment effect could have been due to persistent areas of posterior retinal non-perfusion, even in the laser group, as these areas were too posterior to be safely lasered (37). The RELATE trial is the largest clinical trial to date (*n* = 81) investigating the role of targeted scatter laser photocoagulation for CME in RVOs. This study also did not show any significant reduction in ranibizumab injections with the addition of targeted laser photocoagulation (38). Comparison of all these studies is limited by differences in terms of study population, and laser treatment timing and protocol. As such, the role of targeted scatter laser in RVOs with recalcitrant CME is still unclear. Nevertheless, UWF FA is still clearly useful for assessment and prognostication in these eyes.

### 2.4. Accurate quantification of non-perfusion and ischemia on UWF FA

Much of the research and potential clinical utility of UWF FA in RVOs centers around the accurate quantification of areas of non-perfusion or retinal ischemia. As outlined above, such quantification has been done with different methods, with some approaches focusing on ISI, while others quantify areas of non-perfusion in absolute surface area or disc areas (25–27). The reliability of ISI grading on UWF FA images in terms of intergrader and intragrader agreement has been shown to be acceptable (39). However, one major limitation of UWF images in this regard that has to be acknowledged is the phenomenon of peripheral image distortion and warp. The retina is a three-dimensional (3D) structure that is being imaged, and UWF imaging platforms are able produce a two-dimensional (2D) image of this 3D structure via a mathematical transformation known as stereographic projection (40). This technique produces 2D images where the relative directions and locations of anatomic structures are preserved, but results in unequal magnification and distances, particularly in the periphery. Objects in the peripheral retina appear larger than they are, and the same number of pixels on the 2D image represent a much smaller area in the peripheral retina than in the central/posterior retina (40). This is potentially problematic for approaches that rely on quantifying pixels on an UWF image, such as the ISI, which is defined as the ratio of the number of pixels in non-perfused areas to the number of pixels in the total gradable area of the retina (26, 39).

To address this issue, some groups have used stereographic projection software to compensate for the image distortion, and to provide anatomically accurate estimates of retinal surface area from UWF images in mm^2^ units (40, 41). Interestingly, when uncorrected ISI values and corrected perfusion percentages (based on surface area corrected with stereographic projection) were compared in the same eyes, both measures showed a very high degree of correlation (Spearman correlation *R* = 0.978), and the mean difference between the two measures was low, at 1.4%. However, the authors did point out that the absolute difference could be as high as 14.8% in some cases (40). The accuracy of these corrected measurements has been verified with UWF images of patients with retinal prosthesis implants *in situ*, using the known dimensions of these implants as “ground truth” measurements (42). This stereographic projection software has been incorporated into commercially available UWF devices such as the Optos (Optos PLC) for clinical and research use (43).

### 2.5. Other drawbacks of UWF FA

In spite of the tremendous improvements in imaging technology, UWF imaging and UWF FA in particular still have major drawbacks that need to be acknowledged (Table 1). First, UWF FA is an improvement over standard FA in terms of field of view and the retinal surface area that can be imaged, but it is still fundamentally similar in requiring the administration of intravenous fluorescein dye. This is invasive and time-consuming in clinical practice, and carries small but not insignificant systemic risk. Second, even though more of the periphery can be imaged, the amount of peripheral retina that is imaged varies between eyes, and even between captures of the same eye. Some of this may be due to eyelid or lash artefacts. For qualitative evaluation this may not be so crucial, but it can be a major challenge for assessing quantitative metrics such as ISI over time, which rely on the area of imaged/gradable retina (26, 44). If the area of gradable retina varies in the same eye over time, it can be difficult to differentiate changes in ISI due to changes in actual areas of non-perfusion, versus changes in areas of gradable retina. Third, there have been some discrepancies reported in the assessment of ischemic areas between UWF FA and OCTA, and it is thought that changes in choroidal background fluorescence on UWF FA may account for some of these inaccuracies (45). Fourth, though there have been many attempts to provide greater objectivity and quantification in the evaluation of UWF FA metrics, most of these methods are still very manual and time-consuming, and at the moment are ill-suited to direct clinical application. There has been some preliminary work in automating the identification and quantification of areas of non-perfusion on UWF FA images in DR with artificial intelligence and deep learning, but these will need further validation, particularly if they are to be translated for use in RVOs and other retinal vascular diseases (46, 47). Finally, much of the work on UWF FA and RVOs, particularly efforts to identify clinically-useful thresholds for “ischemic” RVOs has been on retrospective datasets (25–27). Other work linking UWF FA abnormalities to CME and other related outcomes has been largely cross-sectional (29–31). There is a pressing need for more prospective, longitudinal natural history studies with UWF FA in RVO, so that clinically-useful thresholds and cut-offs can be determined and robustly validated.

## 3. Optical coherence tomography angiography

OCTA is a non-invasive imaging modality that allows for evaluation of the retinal microvasculature without the need for invasive dye administration. Based on OCT technology, OCTA infers red blood cell flow by detecting OCT signal changes across multiple, rapidly-acquired, successive OCT scans, and then uses algorithms to derive depth-resolved images of the retinal microvasculature (13, 48). OCTA has a number of key advantages over traditional dye-based angiography techniques. It does not require intravenous dye administration and therefore has no risk of systemic adverse events, it is faster to acquire, it allows superior visualization of the capillary microvasculature, and it provides depth-resolved angiographic images that can be separately segmented to isolate different vascular plexuses in different retinal layers (13, 48). Unlike FA, OCTA technology cannot currently provide information on vascular leakage, though in certain situations this is an advantage, as dye leakage will not obscure vascular or capillary details. Commercial OCTA platforms are based either on spectral domain OCT (SDOCT) or swept source OCT (SSOCT) technology, and currently provide fields of view ranging from 3 × 3 mm to 12 × 12 mm, with larger views possible through image montage. For example, the PLEX Elite 9000 device (Carl Zeiss Meditec, Inc., Dublin, CA, United States) is able to montage five steered 12 × 12 mm OCTA scans into a “panoramic” OCTA image, which has been estimated to cover about 37% of the total retinal surface area, or about half that of an UWF FA image (49).

### 3.1. Qualitative and quantitative vascular abnormalities in RVO

**Figure 3:**
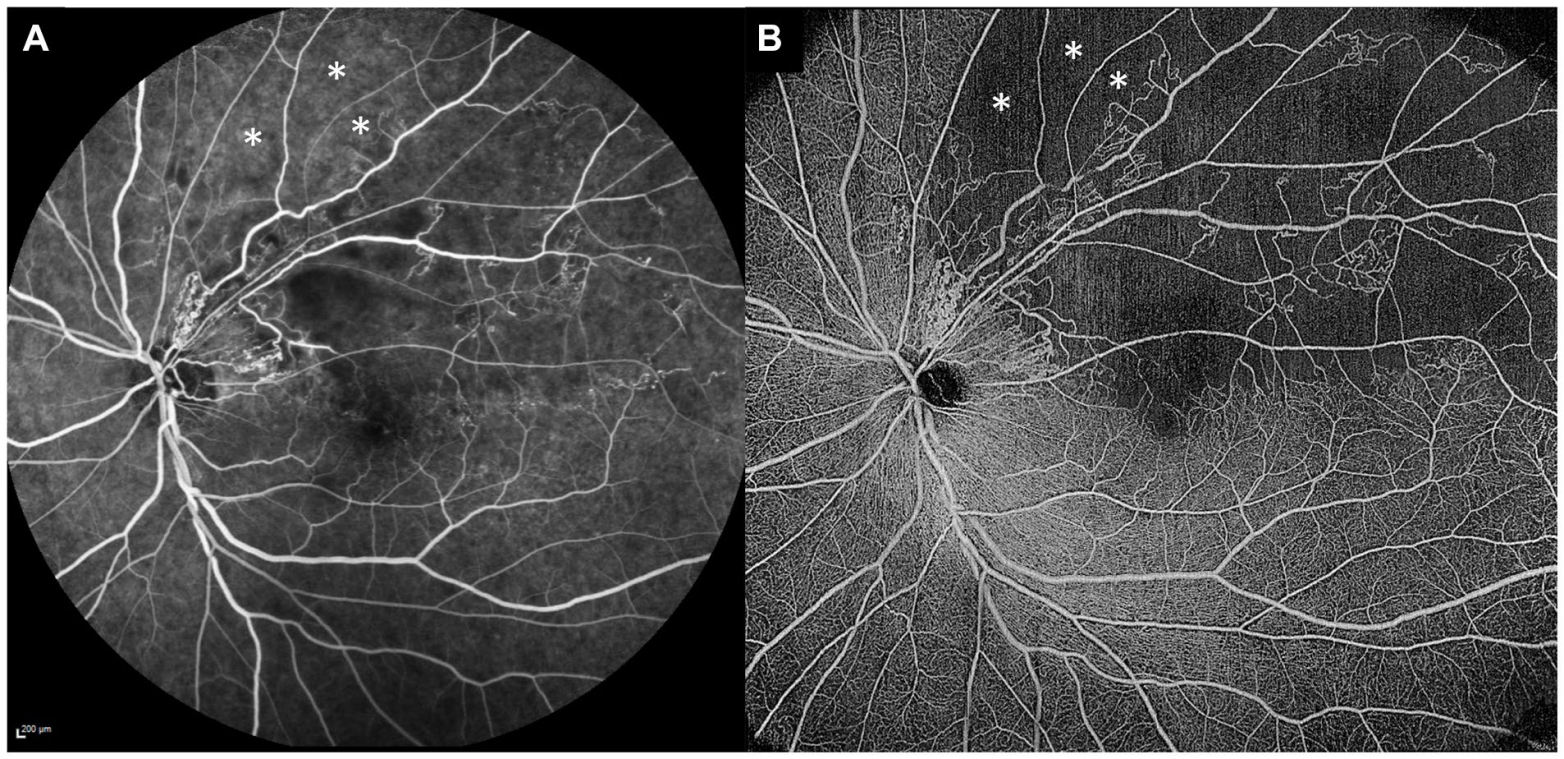
Comparison of standard fluorescein angiography (FA) and optical coherence tomography angiography (OCTA) in the same eye. **(A)** Standard 55° FA image of a superotemporal branch retinal vein occlusion. **(B)** 15 × 15 mm montaged OCTA image of the same eye, demonstrating more extensive areas of retinal non-perfusion than the FA image. The OCTA image clearly demonstrates some areas of retinal non-perfusion just beyond the superotemporal arcade (white asterisks), whereas the same areas on the FA image (white asterisks) are not so well-appreciated as non-perfused, due to the underlying increased choroidal background fluorescence.

Many qualitative vascular abnormalities are evident on OCTA in RVOs. Most of these abnormalities are also ophthalmoscopically visible, such as vascular tortuosity and dilatation, collateral vessels, neovascularization and microaneurysms, but they are often more easily appreciated on OCTA. Other abnormalities are only visible with angiographic techniques, such as capillary non-perfusion and FAZ abnormalities (Figure 3). Multiple observational studies have shown that the qualitative vascular abnormalities demonstrated on OCTA correlate well with the “gold standard” FA (50–52).

Qualitative vascular abnormalities that can be appreciated on OCTA include:

1. Capillary non-perfusion: Seen as areas devoid of visible perfused capillaries. Capillary non-perfusion in RVO is typically more extensive in the deep capillary plexus (DCP) than the superficial capillary plexus (SCP) (13, 53–55).
2. Vascular tortuosity, dilatation and telangiectasias: Affects both the venules and capillaries.
3. Collateral or shunt vessels: These can be either a large vessel traversing an area of non-perfusion, or a group of tortuous vessels near the edge of an area of non-perfusion.
4. FAZ enlargement and irregularity.
5. Microaneurysms.
6. Intraretinal hemorrhages.
7. Cystoid spaces/CME.
8. Retinal neovascularization.
9. Optic disc collaterals.
10. Optic disc neovascularization.

Besides qualitative vascular evaluation, commercial OCTA devices also provide a variety of quantitative vascular metrics related to vessel density, fractal dimension (FD), and foveal avascular zone (FAZ) characteristics such as size, diameter and circularity. Most of these quantitative metrics are provided automatically by commercial devices. Multiple cross-sectional studies have demonstrated that eyes with RVO have significant reductions in capillary vessel density in both the SCP and the DCP (56–60). FD is a quantitative metric that reflects complexity of a branching network, and Koulisis showed reductions in FD in both BRVO and CRVO eyes (60). FAZ changes are also clearly evident in eyes with RVO. Adhi et al. showed that eyes with CRVO had larger FAZ areas compared to BRVO, and both CRVO and BRVO had larger FAZ areas than control eyes (53). Other cross-sectional studies have consistently demonstrated increased FAZ areas and diameters in eyes with RVO compared to healthy controls (57–59, 61, 62). FAZ circularity indices have also been found to be reduced in RVO eyes (56).

Although these quantitative parameters are automated and can be easily obtained from commercial devices, one major drawback that limits their clinical utility is that there are multiple commercial OCTA devices available, and these quantitative metrics are not directly comparable across devices. Furthermore, while significant reductions in RVO eyes can be demonstrated, these are typically not necessary for diagnostic purposes. In the future, they may be useful for prognostication of clinical outcomes such as visual acuity or CME, but with the large majority of studies being cross-sectional in nature, clinically useful cut-off values have yet to be determined and validated.

### 3.2. OCTA and visual acuity

In the absence of neovascularization causing vitreous hemorrhage, tractional retinal detachment or neovascular glaucoma, the major causes of decreased visual acuity in RVOs are related to CME and macular ischemia. CME is readily detected by OCT, and can be treated with intravitreal anti-VEGF agents or corticosteroids. However, visual prognosis after treatment quite frequently depends on the presence and severity of macular ischemia, as well as photoreceptor loss or atrophy. Macular ischemia can only be confirmed with angiography—traditionally with FA, but now with OCTA as well. Typically in OCTA this is seen as an enlarged, irregular FAZ. In fact, OCTA may be the preferred modality for FAZ assessment, as there is no obscuration of detail from dye leakage, which occurs in FA.

A number of studies have looked more closely at the relationship between quantitative metrics and visual acuity in RVOs. Some have shown that poorer visual acuity is correlated with decreased vessel density, decreased FD, and increased FAZ diameter in the DCP, as well as vessel density and FAZ size in the SCP (58, 59, 63, 64). However, many of these studies included a significant proportion of eyes with concurrent CME, and so it is possible that this confounds the analysis and associations (58, 59, 63, 64). Nevertheless, there have been a few studies that excluded eyes with CME, and examined the relationship between OCTA metrics and visual acuity in the absence of this potential confounder. These studies have still found significant correlations between visual acuity and OCTA parameters such as FAZ diameter in the DCP, and FAZ size in the SCP (62, 65) This suggests that OCTA metrics can be useful biomarkers for identifying and monitoring macular ischemia, and can be informative for visual prognostication in RVOs.

### 3.3. OCTA and cystoid macular edema

As with UWF FA, various OCTA metrics have also been associated with the presence of persistent CME in RVOs. Given the greater field of view and advantages of UWF FA, these studies have concentrated mainly on the overall extent and location (peripheral vs. posterior) of non-perfusion areas and CME (29–31). In contrast, while OCTA provides a more limited field of view, this modality has the key advantage of providing depth-resolved analysis of the different vascular plexuses, including the SCP and DCP. This has allowed for analysis of relative differences between perfusion areas in the SCP and DCP, and their relation to CME.

OCTA studies have observed that RVOs with CME demonstrate areas of decreased or absent flow in the DCP, and that CME tends to recur in these regions (54, 66). It has been postulated that these areas of absent flow or “perfusion gaps” in the DCP affect intraretinal fluid management, and may therefore contribute to the occurrence or persistence of CME (66). A few studies have examined this question retrospectively. Tsuboi et al. identified areas with “gap vessels” where there was selective DCP loss, by subtracting DCP vessel images from the corresponding SCP vessel images (67). In 20 eyes with BRVO, they showed that areas with gap vessels were significantly larger in eyes that had persistent CME, compared to those without. In a similar vein, Bae et al. evaluated perfusion gaps in 19 eyes with BRVO and CME, and also concluded that larger perfusion gaps (on 12 × 12 mm OCTA scans) were associated with greater anti-VEGF treatment burden for CME (68). Yeung et al. used a slightly different metric, by quantifying deep-superficial flow ratio (DSFR), which was calculated by dividing DCP vessel density by SCP vessel density (69). They showed in 30 eyes with BRVO that DSFR was significantly lower in eyes with refractory CME, compared to those with a better treatment response. One potential concern with this approach is that the temporal and causative relationships between DCP perfusion gaps and areas of CME have not been definitively established. It is currently not clear whether perfusion gaps occur first and lead to CME, or whether areas of CME develop first (e.g., due to vascular leakage or other mechanisms) and result in displacement of DCP vessels forming “gaps.” This highlights the need for longitudinal studies in this area.

### 3.4. Non-perfusion on OCTA

As outlined above, detection of significant areas of retinal non-perfusion is one of the main clinical indications for performing FA or UWF FA in RVOs. As with FA, areas of retinal non-perfusion can also be detected and quantified on OCTA. Table 1 summarizes the advantages and disadvantages of using OCTA for the detection of retinal non-perfusion. FA has been the gold standard for determination of retinal non-perfusion, and it has been important to establish that areas of non-perfusion on OCTA match those on FA. Most studies examining this question in retinal vascular diseases have shown that there is good and substantial agreement between FA and OCTA (70–73). Firstly, in DR, Sawada et al. showed that OCTA (12 × 12 mm scans) could detect areas of non-perfusion qualitatively as well as UWF FA (70). Similarly, in a cohort of eyes with DR, Hirano et al. also showed very high levels of agreement for non-perfusion between 12 × 12 mm OCTA scans and FA (72). In that study, OCTA detected areas of non-perfusion with 95% sensitivity and 100% specificity, and when they compared the quantified areas of non-perfusion between the two modalities, they were highly concordant (72). There is also good evidence to support that this correlation is true in RVO eyes as well. Kadomoto et al. showed that areas of non-perfusion correlated well between OCTA and UWF FA in BRVO (73). Shiraki et al. also examined a cohort of eyes with BRVO who had both OCTA and UWF FA performed. Within the same areas that were imaged on both modalities, they found excellent correlation in non-perfusion areas quantified by the two modalities (*R*^2^ = 0.9429, *p* < 0.0001) (71). To our knowledge, there has been one report of significant discrepancies between OCTA and UWF FA in non-perfusion areas in DR (45). In this study, the authors examined both OCTA and UWF FA images before and after intravitreal anti-VEGF treatment, to see if there were significant changes in retinal perfusion. There were apparent areas of re-perfusion on UWF FA, but OCTA in the same areas demonstrated clearly that this was not the case. The authors attributed this spurious re-perfusion on UWF FA as likely due to changes in choroidal background fluorescence, and suggested therefore that OCTA may be a more accurate modality for quantifying retinal non-perfusion than FA (45). Figure 3 shows paired standard FA and OCTA images from the same eye with a BRVO, demonstrating this phenomenon, where clear areas of non-perfusion on the OCTA image are easily missed on FA, due to increased underlying choroidal background fluorescence.

**Figure 4:**
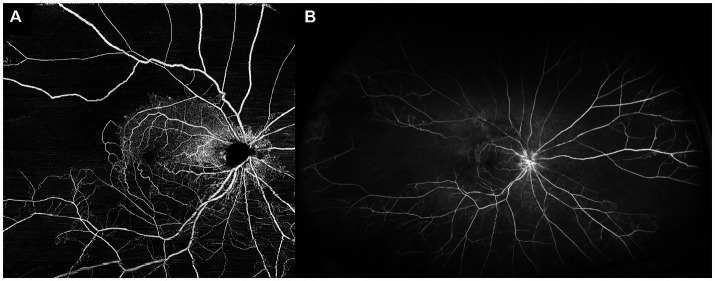
Comparison of optical coherence tomography angiography (OCTA) and ultra-widefield fluorescein angiography (UWF FA) in the same eye. **(A)** 15 × 15 mm montaged OCTA image of a central retinal vein occlusion, allowing for visualization of the posterior pole and some of the mid-peripheral retina. **(B)** UWF FA image of the same eye, showing a much wider field of view, and demonstrating significant areas of peripheral retinal non-perfusion that were not detectable with the field of view of the OCTA image.

While OCTA may be more convenient, faster, and potentially more accurate for quantification of retinal non-perfusion in comparison to FA, the limitation of current OCTA technology is in field of view. Current commercial OCTA platforms are able to image up to 12 × 12 mm scans, and can cover larger areas with image montage, but this field of view is still much less than UWF FA is able to achieve (Figure 4). Nevertheless, many groups have attempted to see if OCTA of the posterior retina is able to infer peripheral retinal non-perfusion as well. These studies have generally shown that OCTA metrics and non-perfusion from the posterior retina correlate well with peripheral non-perfusion as revealed by UWF FA (64, 74–77). Huang et al. showed that non-perfusion areas on a 3 × 3 mm OCTA scan correlated well with non-perfusion area (*R* = 0.688, *p* < 0.01) and ISI (*R* = 0.680, *p* < 0.01) on UWF FA (75). Cavalleri et al. similarly showed that OCTA FAZ area (*R* = 0.63, *p* = 0.019), and vessel density in the SCP (*R* = −0.62, *p* = 0.022) and DCP (*R* = −0.66, *p* = 0.011), all correlated significantly with ISI on UWF FA (77). Ryu et al. looked at multiple OCTA parameters such as vessel density and FD from the SCP and DCP on 6 × 6 mm OCTA scans and demonstrated that they all correlated with ISI on UWF FA. When attempting to use OCTA parameters to detect “severe retinal ischemia” (defined as ISI > 10%), all the OCTA parameters achieved an area under the receiver operating characteristic curve (AUC) of >0.9, with FD in the DCP showing the greatest classification performance (AUC = 0.948). Based on their series, they were also able to suggest a cut-off of 5.39% for FD, which would perform well in detection of “severe retinal ischemia” (74). Glacet-Bernard et al. showed that vessel density in the SCP and DCP on 12 × 12 mm OCTA scans correlated significantly with ISI on UWF FA, and that OCTA had 100% sensitivity and 64% specificity for detection of “marked nonperfusion” (defined as ISI ≥ 25%) on UWF FA (76).

The evidence from these studies suggests that although the extent of peripheral retina that can be assessed with OCTA is currently limited, OCTA of the posterior/central retina can still reliably infer areas of non-perfusion in the retinal periphery. Most of these authors suggest therefore, that OCTA can be used as a non-invasive, convenient “screening tool,” to identify those eyes who are at risk of having significant peripheral non-perfusion, which would then benefit from an UWF FA procedure.

### 3.5. OCTA and neovascularization

Similar to how the definition of an “ischemic” RVO needs to be re-examined in UWF FA, some groups have attempted to try and re-define “ischemic” RVOs using OCTA, though this work is still in its initial stages. An et al. performed a cross-sectional study on eyes with CRVO, where they classified them into ischemic or non-ischemic based on areas of non-perfusion on FA (78). They then showed that ischemic CRVOs had lower SCP and DCP vessel densities and larger FAZ area on 3 × 3 mm OCTA scans. They suggested that DCP vessel density was the best parameter for classification, with an AUC of 0.962, and with a threshold of ≤38.4%, DCP vessel density achieved 100% sensitivity and 92.3% specificity for classifying ischemic CRVOs. Khodabandeh et al. used OCT 3 × 3 mm and 8 × 8 mm OCTA scans to classify CRVOs as ischemic, based on the presence of a relative afferent pupillary defect and visual acuity worse than 20/200. Ischemic CRVOs by this definition had lower SCP and DCP vessel densities, and their best-performing classification model had an AUC of 0.84, with 100% sensitivity and 64% specificity (79). The major drawback to both these studies is that their definitions of “ischemic” CRVOs were cross-sectional, and based on imperfect ground truth classifications. Ideally, this question should be investigated with a longitudinal natural history study, with baseline OCTA metrics, and longitudinal observation for neovascularization and associated complications. Kadomoto et al. undertook a small longitudinal cohort study of 26 patients with treatment-naïve BRVOs, performed “baseline” OCTA after 3 monthly anti-VEGF injections, and followed them prospectively for the development of neovascularization over another 9 months (73). They reported that larger non-perfusion areas on OCTA were associated with the development of neovascularization. Similar longitudinal studies, on a larger scale, will be necessary to re-define “ischemic” RVOs using baseline OCTA metrics. Nevertheless, this remains a promising approach. More recently, an international expert consensus group on OCTA imaging published a report recommending that OCTA can be used to define an “ischemic” CRVO, and suggested that because OCTA fields of view vary among different devices, that such definitions should be based on a percentage of the absolute imaged area in which there is “no flow” or non-perfusion (80). They further suggested that ≥30% of “no-flow area” be used to define an ischemic CRVO, though this was a recommendation, and not based on actual cross-sectional or longitudinal data defining risk of neovascularization. This definition will need to be validated in future studies.

OCTA can also be very useful for diagnosis of retinal neovascularization in select cases. Retinal neovascularization is defined as a vascular structure with demonstrable flow anterior to the internal limiting membrane (ILM), and it has been shown that OCTA is very useful for diagnosis of neovascularization and differentiating them from intraretinal microvascular abnormalities (IRMAs) in DR (81). Arya et al. reported that OCTA had 92% sensitivity and 99% specificity for differentiating neovascularization from IRMAs in DR (81). Similarly in RVOs, there may be some suspicious collateral vessels (which by definition are intraretinal, and do not cross the ILM), that can be clinically difficult to differentiate from neovascularization, and OCTA can be an effective, convenient and non-invasive tool in this scenario. Sakimoto et al. reported a case where OCTA effectively clinched the diagnosis of neovascularization after a BRVO (82). In their case, OCTA demonstrated definite retinal neovascularization from about 6 months after presentation, which was not evident on FA. On FA there was some diffuse hyperfluorescence that looked like apparent retinal re-perfusion, without significant leakage. This is an example of a case where OCTA was the better imaging modality for diagnosing neovascularization.

In cases of established retinal neovascularization, OCTA provides much better cross-sectional detail and structural characterization than FA. Sogawa et al. published an example of this, where OCTA demonstrated a retinal neovascular membrane after a BRVO more clearly than FA, and could provide detailed structural information (83). They were able to show with OCTA that the outer border of the neovascularization consisted of looping radial peripapillary capillaries, and that the posterior hyaloid was firmly adherent to the neovascularization, which may have further prognostic and management implications. Huemer et al. looked at a larger series of retinal neovascularization after ischemic RVOs, and could on the basis of OCTA structure, classify the neovascular tufts into different phenotypic types, such as sea-fan or nodular types (84). They found in their series of ischemic RVOs that OCTA had a much higher detection rate for neovascularization than clinical examination, and even detected one case that was missed on UWF FA. That lesion was of the nodular type, which the authors suggest can be easily mistaken for hemorrhage on clinical examination, and which is difficult to detect on FA because they may not leak significantly. Though classification of neovascularization is now possible with OCTA into different structural phenotypes, the clinical implications and outcomes of these different phenotypes are as yet unclear. OCTA could also be used to follow longitudinal changes in neovascular tufts after treatment to determine regression or the presence of persistent flow, though the clinical implications of these would need to be similarly validated (84, 85). Table 1 summarizes the main advantages of using OCTA to detect neovascularization.

### 3.6. Drawbacks of OCTA

OCTA technology is clearly promising, and has the potential to provide important angiographic information in a non-invasive manner, to inform prognosis and management of retinal vascular diseases. Nevertheless, this technology has important limitations and drawbacks, some of which have been discussed above (Table 1). The first, and arguably most important limitation, is the heterogeneity and lack of standardization in the field (80, 86). There are multiple different commercial OCTA platforms, using different proprietary algorithms, and providing different quantitative metrics, which are not interchangeable. There are currently no well-established, standardized guidelines for studies reporting OCTA metrics or outcomes. These factors significantly limit the reproducibility and quality of the evidence available for OCTA, which in turn limits the incorporation of OCTA into daily clinical practice and decision-making. There are ongoing efforts among international expert consensus groups to address this issue, and we can expect that more standardized guidelines and nomenclature will be forthcoming soon (80, 86). Second, it is evident from this review that the large majority of evidence for the use of OCTA in RVOs is based on cross-sectional studies. Prospective, longitudinal studies in this area are few, but they are needed for robust validation of OCTA metrics and cut-offs for clinical use. Third, there are still technological limitations, such as issues with image artefacts, quality and gradability issues, and limited field of view. However, OCTA technology is improving rapidly, and we expect that the impact of these technological limitations can be minimized with time.

## 4. Conclusion

Imaging technology such as UWF FA and OCTA clearly provide a wealth of new information over standard FA imaging techniques, that has the potential to improve clinical management in patients with RVO. Certain key characteristics of these imaging platforms are important advantages for clinical utility—such as the evaluation of a much larger area of the retinal periphery for UWF FA, and the non-invasive, quantitative nature of OCTA. Consequently, by providing more information on perfusion in the peripheral retina, UWF FA in clinical practice has the potential to improve risk stratification and prognostication for neovascularization and CME in RVOs, without any major added drawbacks over standard FA technology. OCTA provides non-invasive information on retinal perfusion, which can inform the need for more invasive dye-based angiography, and also has the potential to provide effective prognostication of important clinical outcomes, such as neovascularization, CME, and eventual visual acuity. Major unmet needs in the field are that of standardization and validation of clinically useful cut-offs with prospective, longitudinal data. These imaging tools have already proved useful in clinical practice for management of RVOs, and as more evidence becomes available to guide our management, they will continue to further improve clinical outcomes for patients with RVO.

## Author contributions

T-ET and KT contributed to conception of the study. T-ET, FI, PC, and KT contributed to drafting and revising the manuscript. All authors contributed to the article and approved the submitted version.

## Conflict of interest

The authors declare that the research was conducted in the absence of any commercial or financial relationships that could be construed as a potential conflict of interest.

## Publisher’s note

All claims expressed in this article are solely those of the authors and do not necessarily represent those of their affiliated organizations, or those of the publisher, the editors and the reviewers. Any product that may be evaluated in this article, or claim that may be made by its manufacturer, is not guaranteed or endorsed by the publisher.
